# Strontium ranelate promotes chondrogenesis through inhibition of the Wnt/-catenin pathway

**DOI:** 10.1186/s13287-021-02372-z

**Published:** 2021-05-20

**Authors:** Hao Yu, Yan Liu, Xiangwen Yang, Jiajing He, Fan Zhang, Qun Zhong, Xiaojing Guo

**Affiliations:** 1 Department of Prosthodontics, Shanghai Stomatological Hospital, Fudan University, Shanghai, 200001 China; 2 Shanghai Key Laboratory of Craniomaxillofacial Development and Diseases, Fudan University, Shanghai, 200001 China; 3 Department of Orthodontics, Shanghai Stomatological Hospital, Fudan University, Shanghai, 200001 China

**Keywords:** Strontium ranelate, BMSCs, Chondrogenesis, Cartilage regeneration, Wnt/-catenin pathway

## Abstract

**Background:**

Cartilage regeneration is a key step in functional reconstruction for temporomandibular joint osteoarthritis (TMJ-OA) but is a difficult issue to address. Strontium ranelate (SrR) is an antiosteoporosis drug that has been proven to affect OA in recent years, but its effect on chondrogenesis and the underlying mechanism are still unclear.

**Methods:**

Bone mesenchymal stem cells (BMSCs) from SpragueDawley (SD) rats were induced in chondrogenic differentiation medium with or without SrR, XAV-939, and LiCl. CCK-8 assays were used to examine cell proliferation, and alcian blue staining, toluidine blue staining, immunofluorescence, and PCR analysis were performed. Western blot (WB) analyses were used to assess chondrogenic differentiation of the cells. For an in vivo study, 30 male SD rats with cartilage defects on both femoral condyles were used. The defect sites were not filled, filled with silica nanosphere plus gelatine-methacryloyl (GelMA), or filled with SrR-loaded silica nanosphere plus GelMA. After 3months of healing, paraffin sections were made, and toluidine blue staining, safranin O/fast green staining, and immunofluorescent or immunohistochemical staining were performed for histological evaluation. The data were analyzed by SPSS 26.0 software.

**Results:**

Low concentrations of SrR did not inhibit cell proliferation, and the cells treated with SrR (0.25mmol/L) showed stronger chondrogenesis than the control. XAV-939, an inhibitor of -catenin, significantly promoted chondrogenesis, and SrR did not suppress this effect, while LiCl, an agonist of -catenin, strongly suppressed chondrogenesis, and SrR reversed this inhibitory effect. In vivo study showed a significantly better cartilage regeneration and a lower activation level of -catenin by SrR-loaded GelMA than the other treatments.

**Conclusion:**

SrR could promote BMSCs chondrogenic differentiation by inhibiting the Wnt/-catenin signaling pathway and accelerate cartilage regeneration in rat femoral condyle defects.

## Background

Temporomandibular joint osteoarthritis (TMJ-OA) is a joint degenerative disease characterized by cartilage lesions along with changes in the synovium and degradation of subchondral bone. Epidemiological surveys showed that the prevalence of TMJ-OA was 25% in the 2049year age group [1]. The main symptoms of TMJ-OA are joint friction, joint pain, stiffness, and functional limitation. Histologically, abnormal synthesis and degradation of articular chondrocytes, extracellular matrix, and subchondral bone are detected; as a result, the cartilage becomes thinner and stripped, and subchondral bone is exposed and scleroid [2]. The pathogenesis of TMJ-OA involves inflammation, excessive mechanical stress, abnormal remodeling of subchondral bone, chondrocyte apoptosis, catabolic disturbances, and genetic factors, in which accelerated subchondral bone turnover plays a role in the initiation of TMJ-OA [3]. Subchondral osteosclerosis can lead to dysfunction of the cartilage tissue and cartilage degeneration; conversely, cartilage degeneration can also lead to subchondral bone metabolic disorders, subchondral bone remodeling, and sclerosis [4, 5]. This vicious cycle strongly promotes the development of OA. Inhibition of this vicious cycle and regeneration of the degraded or even exfoliated cartilage are key to the treatment of OA and the reconstruction of joint function.

For the treatment of TMJ-OA, conventional treatments, including physical therapies, occlusal splints, and anti-inflammatory medications, are often ineffective. Surgical intervention involving joint replacement or cartilage regeneration is important in the clinic [6]. Cartilage regeneration is a key step of functional reconstruction, especially for TMJ-OA, in which the condyle, as the center of mandibular growth and development, is critical in regulating the length and width of the whole mandible. However, cartilage tissue regeneration is also a major challenge, because it is expensive and has limited clinical effects. Due to the poor proliferation of chondroblasts and the lack of blood supply in cartilage tissue, it is difficult for stem cells and chondroblasts to migrate, proliferate and differentiate in defect sites. As research on stem cell therapy has matured, tissue engineering technology with mesenchymal stem cells (MSCs) as seed cells has shown promise for cartilage damage repair [7]. One of the major challenges of stem cell therapy is to control the differentiation of stem cells into chondrocytes. In the past, cytokines were used. Although is highly effective, a great amount of growth factors needs to be applied locally for a long time. The technical requirements and economic cost of obtaining growth factors are high, and long-term release is difficult, which substantially limits the clinical application of this method.

Strontium ranelate (SrR), as an antiosteoporosis drug, can effectively promote bone formation and suppress bone resorption, and its anti-OA effect has been confirmed in recent years. The Strontium Ranelate Efficacy in Knee Osteoarthritis (SEKIOA) phase III clinical trial has provided the most reliable clinical evidence. In this 3-year multicenter randomized controlled trial, the SrR-treated patients showed notable pain relief, functional improvement, and radiological amelioration compared with the placebo-treated patients [8, 9]. SrR is considered a disease-modifying OA drug (DMOAD) that is used to avoid or delay surgery [10,12]. However, although strong clinical evidence of the anti-OA effect of this drug has been published, the underlying mechanism of SrR is still unclear. Currently, most studies have reported that the anti-OA efficacy of SrR is mediated through the inhibition of subchondral bone resorption via the OPG/RANK/RANKL system [8,13]. It also shows anti-inflammatory effects, especially suppress the expression of matrix metalloproteinases (MMPs). Tat found that SrR could downregulate the mRNA levels of MMP-2 and MMP-9 [14], and Pelletiers study showed that SrR downregulated the expression levels of MMP-1, MMP-13, and cathepsin K, thus suppressing bone/cartilage resorption [15].

Strontium ions have a similar molecular structure to calcium ions and may have similar physiological functions. After SrR acts on the Ca^2+^ ion receptor on the cell membrane surface, it activates the canonical or noncanonical Wnt pathway through the calcineurin-activated T nuclear factor (Cn-NFAT) signaling pathway. Fromigue et al. confirmed that the positive effect of SrR on the Wnt pathway can promote osteogenic differentiation [16]. However, no studies have examined the effect of SrR on cartilage differentiation, and no experimental evidence in vivo has shown the effect of local SrR administration on cartilage regeneration. Therefore, we selected the silica nanosphere, which is also called mesoporous silica nanoparticles (MSNs) as the drug carrier, and combined with gelatine methacryloyl, GelMA scaffold to achieve a local control release of SrR. GelMA is a novel scaffold with good biocompatibility, good biodegradability, and easy to operate [17]. Considering the relative low bioactivity, weak mechanical property, and weak interaction with the cells of scaffolds alone, we added silica nanospheres to enhance the function. Silica nanospheres are widely used in tissue engineering as a drug carrier, with high specific surface area, high loading capacity, good biocompatibility, and bioactivity in cartilage tissue [18]. The combination of these two biomaterials provided us a good way of local drug administration.

The main purpose of the present study was to investigate whether SrR has a positive effect on the chondrogenic differentiation of BMSCs and assess the hypothesis that SrR regulates the chondrogenic differentiation of BMSCs through the Wnt/-catenin signaling pathway. Then, a cartilage defect model was generated in rat femurs, and a local sustained-release administration method by silica nanospheres plus GelMA scaffold was used to determine whether the local application of SrR can promote cartilage tissue regeneration. The results may provide an alternative for drug application in cartilage tissue engineering.

## Methods

### Isolation and culture of rat BMSCs

Male SD rats at 46weeks of age were purchased from Vital River Laboratories (Shanghai, China) and sacrificed by an overdose of pentobarbital sodium. After the removal of all of the soft tissue from the femurs, the marrows were collected from both ends of the femurs and seeded on cell plates to obtain BMSCs. BMSCs at passages 24 were used for the present study.

All animal treatments were strictly in accordance with NIH guidelines and were approved by the Animal Research Committee of the Shanghai Stomatological Hospital and Shanghai Research Centre of Model Animal Organization (IACUC No. 2020-0010-06).

### Treatment with SrR and chondrogenic differentiation medium

51.35mg *SrR* (Sigma-Aldrich, USA) was dissolved in 50mL of liquid to obtain the highest concentration of 2.0mmol/L and diluted to various concentrations as previously described [19].

The chondrogenic differentiation medium was prepared as described by Solchaga [20]. DMEM basic medium (Gibco, 11965175) contained 10ng/mL TGF-3 (Prospec, CYT-886), 100U penicillin/streptomycin (Gibco, 15140163), 1% fetal bovine serum (Gibco, 10099), 10^7^mol/L dexamethasone (Sigma, 50-02-2), 50mg/LL-ascorbic acid (Sigma, A4403), and 1 ITS-A (Gibco, 51300-044) were used.

### Cell proliferation assay of BMSCs

CCK-8 assay was used to assess cell proliferation of BMSCs treated with different concentrations of SrR. Briefly, BMSCs were seeded in 96-well plates (starting cell density of 3 10^3^ cells/well) and treated with different concentrations of SrR (0.125, 0.25, 0.5, 1.0, and 2.0mmol/L) for 1, 3, 5, and 7days. Then, 10L of CCK-8 solution was added to each plate and incubated in the dark in a 5% CO_2_ and 37C environment for 1h. Cell viability was measured by a microplate reader (ELX 800; BioTek Instruments, USA) at a wavelength of 450nm.

### Analysis of the Wnt/-catenin signaling pathway

To determine the effect of SrR on the Wnt/-catenin signaling pathway, we used XAV-939 (Selleck, China), an inhibitor of -catenin, at a concentration of 2.0 mmol/L and LiCl (Sigma, USA), an agonist of -catenin, at a concentration of 2.0mmol/L. Grouping was performed as follows: control, 0.25mmol/L SrR, 0.50mmol/L SrR, XAV-939, XAV-939 + 0.25mmol/L SrR, LiCl, and LiCl + 0.25mmol/L SrR. After 14days of induction, alcian blue and toluidine blue staining, immunofluorescence staining, hydroxyproline (Hyp) assays, PCR, and Western blot assays were performed.

### Alcian blue staining and toluidine blue staining

BMSCs were seeded on 24-well plates at an initial density of 1 10^5^ cells/well and changed to chondrogenic differentiation medium with or without SrR, XAV-939, or LiCl. After 14days of induction, the cells were fixed with 4% paraformaldehyde and stained with alcian blue dye solution (Solarbio, China) and toluidine blue dye solution (Solarbio, China). Then, the cells were photographed by inverted light microscopy (Leica DMI 3000B, Germany).

### Immunofluorescence staining assay

BMSCs were cultured in chondrogenic differentiation medium with or without SrR, XAV-939, and LiCl for 14days and fixed with 4% paraformaldehyde for staining. First, the cells were permeabilized with PBST and rinsed with PBS several times. Nonspecific interactions were blocked with goat serum, and the cells were incubated with primary antibodies. Col-II (Novus Biologicals, NB600-844, USA) and MMP-9 (Proteintech, 10375-2, USA) antibodies were used as primary antibodies, Alexa Fluor 488 IgG (Invitrogen, A11001, USA) was used as the secondary antibody, and DAPI (Sigma, D9642) was used for core staining. Cells were photographed by microscopy.

### Hydroxyproline (Hyp) assay

Hyp assay kit (Abcam, ab222941, USA) was used according to the manufacturers instructions. The cell culture supernatants, ddH_2_O, and the standard protein sample were prepared, added to an equal volume of NaOH, evaporated, cooled, and neutralized with an equal amount of HCl. Then, the cells were centrifuged, and the supernatants were collected in a new tube. Oxidation reagent was added and incubated at room temperature for 20min, and the developer was added and incubated at 37C for 5min. Then, DMAB concentrate was added and incubated at 65C for 45min. The OD values of each group were measured at a wavelength of 560nm. Hyp concentrations were calculated by the standard curve.

### Quantitative real-time PCR assay

BMSCs were cultured in 6-well plates and treated with or without SrR, XAV-939, or LiCl for 1, 7, or 14days. Total RNA was extracted from BMSCs using TRIzol reagent (Ambion, USA), followed by reverse transcription to generate cDNA with Tiangen FastKing cDNA Dispelling RT SuperMix (Applied Biosystems, USA) according to the manufacturers instructions. For RNA expression analysis, cDNA (100ng) was amplified in a 20L reaction system containing 10L of 2 SuperReal PreMix Plus, 1.2L of primer, and 6.8L of ddH_2_O. The primer sequences are listed in Table 1. Triplicate reactions were performed, and the relative fold change in gene expression was calculated (LightCycler 96 PCR system, Roche, Germany).

**Table 1:** Primer sequences used for the BMSCs

Gene target	Primer sequence forward(5-3)	Primer sequence reverse(5-3)
Sox-9	TCCCCGCAACAGATCTCCTA	AGCTGTGTGTAGACGGGTTG
Col-II	ATCGCCACGGTCCTACAATG	GGCCCTAATTTTCGGGCATC
MMP-9	GATCCCCAGAGCGTTACTCG	GTTGTGGAAACTCACACGCC
-catenin	ACTCCAGGAATGAAGGCGTG	GAACTGGTCAGCTCAACCGA
Aggrecan	CAAGTCCCTGACAGACACCC	GTCCACCCCTCCTCACATTG
GAPDH	AGTGCCAGCCTCGTCTCATA	GATGGTGATGGGTTTCCCGT

### WB assay

BMSCs were cultured in 6-well plates with chondrogenic differentiation medium with or without SrR, XAV-939, and LiCl for 14days. Total protein was extracted by RIPA buffer and measured by a BCA protein assay kit. Equal amounts of proteins were subjected to 12% SDS-PAGE and transferred to PCDF membranes. After the membranes were blocked with 5% skim milk, they were exposed to the primary antibody, including Sox-9 (Santa Cruz, sc-166505), -catenin (CST, 9562, USA), MMP-9 (Proteintech, 10375-2), aggrecan (Proteintech, 13880-1), and -actin antibodies (CST, 4970), at 4C overnight. Then, the cells were washed with PBST and incubated with HRP-conjugated goat anti-rabbit or rabbit anti-mouse IgG (Beyotime, China) for 2h at room temperature. The membranes were thoroughly washed, and ECL reagent (Pierce, Rockford, IL, USA) was used for visualization. Bands were photographed and measured by the Quantity One analysis system (Bio-Rad).

### Synthesis of SrR-loaded silica nanospheres and GelMA gel

Silica nanospheres were synthesized according to the sol-gel approach as described by a previous study [21]. Briefly, 3.0g ammonium fluoride (NH_4_F) and 1.82g cetyltrimethylammonium bromide (CTAB) were dissolved in ddH_2_O, heated to 80C, and stirred for 1h. Nine milliliters of tetraethoxysilane (TEOS) was added in a dropwise manner, centrifuged (8000rpm, 25min) in the suspension solution, and maintained overnight at room temperature. Then, the sample was freeze-dried for 12h, heated to 600C, and calcined for 6h. For SrR drug loading, the nanospheres were immersed and stirred in PBS containing 2.0mmol/L SrR for 48h. The morphology of the silica nanospheres was observed by scanning electron microscopy (SEM) and characterized by energy dispersive spectrum analysis (EDS). Then, the drug-releasing speed was tested. We added 80L of SrR at a concentration of 2.0mmol/L to 0.1g silica nanospheres, and after it was completely absorbed and dried, these nanospheres were immersed in 2mL of PBS at 37C. In addition, the supernatant was collected and refreshed at 1h, 2h, 4h, 8h, 12h, 1day, 2days, 3days, 4days, 5days, 6days, 7days, 8days, and 9days. The SrR content was quantified by ultraviolet spectrophotometry.

Product of gelatine methacryloyl, GelMA (ELF, EFL-GM-30, Suzhou, China) were used as scaffold carriers of SrR-loaded nanospheres to obtain desirable mechanical properties. In short, gelatine directly reacts with MA in phosphate buffer, and lithium phenyl-2,4,6-trimethylbenzoylphosphinate (LAP) is used as a photoinitiator [22]. The SrR-loaded nanospheres were mixed with GelMA jelly at a concentration of 0.1g/mL and incubated in the dark at 4C for storage.

### Cell viability assay and PCR assay on scaffold

Certain amount of silica nanospheres mixed with GelMA jelly were added to 96-well plates or 6-well plates; the quantity was sufficient to cover entire growth surface and UV curved for 20 s. Then, BMSCs were seed on the solidified gel at the cell density of 3 10^3^ cells/well for 96-well plates and 1 10^6^ cells/well for 6-well plates. CCK-8 assay was conducted to investigate the cell viability on scaffold as previous described. For PCR test, non-loaded silica nanospheres plus GelMA was control group, SrR-loaded silica nanospheres plus GelMA was SrR group, and cultural medium with LiCl upon the SrR-loaded silica nanospheres plus GelMA was SrR+LiCl group. The gene-expressed levels of -catenin and Sox-9 were tested after 1day and 7days of induction.

### Animal surgery

**Fig. 1:**
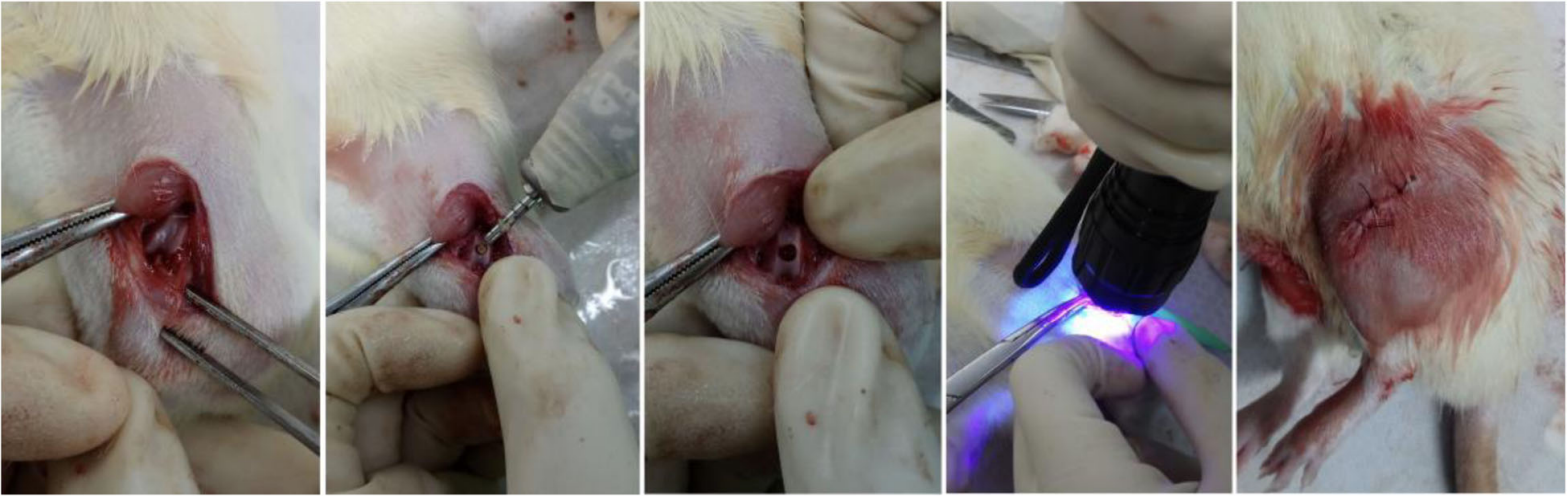
Animal surgical procedures. From the left to the right showed the procedures of animal surgery. After general anesthesia, a cylindrical defect of 2.5mm in width and 1.0mm in depth was made on the center of the trochlea and filled with GelMA gelatine with or without SrR-loaded nanospheres. After curved the materials with UV light, we carefully sutured every layer of the tissues, and animals were fed under normal condition for 3months after surgery

Thirty healthy male SD rats at 89weeks of age were purchased from Vital River Laboratories (Shanghai, China) and fed in SPF conditions for 1week before the surgery. The femoral condyle defect model was established following the procedure of a previous study [23]. Briefly, medial parapatellar arthrotomy was performed on both sides of the knees under general anesthesia, and the femoral trochlea was fully exposed. A round defect with 2.5mm diameter and 1.0mm depth was made on the center trochlea by a trephine under cooling with saline. Cartilage defects were left empty for the empty group (*n*=10) and were filled with GelMA + nanospheres in the control group (*n*=10). The defects in the test group (*n*=10) were filled with GelMA + SrR-loaded nanospheres and cured by UV light for 30s. All of the wounds were carefully sutured, and the muscle layer was reverted back to the original position. The animals were euthanized 3months after the surgery (Fig. 1).

### Histological and immunohistochemical evaluation

Samples were collected and fixed in formalin for 3days, followed by 6weeks of decalcification. Paraffin sections were made and stained with toluidine blue, safranin O/fast green, Sox-9 (Abcam, 185966, UK), MMP-9 (Abcam, ab7003, UK), and -catenin antibodies (Abcam, ab32572, UK) for immunohistochemical staining. Besides, immunofluorescent staining of -catenin was conducted, and photographed by laser scanning confocal microscope (Leica TCS SP 8 X).

The histological characteristics of the regenerated cartilage tissue were scored using the International Cartilage Repair Society (ICRS) II scoring system [24], as shown in Table 2. Toluidine blue-stained and safranin O/fast green-stained sections were assessed by three independent doctors blinded to the grouping, and the average scores were calculated.

**Table 2:** The International Cartilage Repair Society II scoring system [24]

Histological parameter	Score (0100)
1. Tissue morphology	0: Full thickness collagen fibers 100: Normal cartilage birefringence
2. Matrix staining	0: No staining 100: Full metachromasia
3. Cell morphology	0: No round/oval cells 100: Mostly round/oval cells
4. Chondrocyte clustering	0: Present 100: Absent
5. Surface architecture	0: Delamination or major irregularity 100: Smooth surface
6. Basal integration	0: No integration 100: Complete integration
7. Formation of a tidemark	0: No calcification front 100: Tidemark
8. Subchondral bone abnormalities/marrow fibrosis	0: Abnormal 100: Normal marrow
9. Inflammation	0: Present 100: Absent
10. Abnormal calcification/ossification	0: Present 100: Absent
11. Vascularization (within the repaired tissue)	0: Present 100: Absent
12. Surface/superficial assessment	0: Total loss or complete disruption 100: Resembles intact articular cartilage
13. Mid/deep zone assessment	0: Fibrous tissue 100: Normal hyaline cartilage
14. Overall assessment	0: Bad, fibrous tissue 100: Good, hyaline cartilage

### Statistical analysis

Data are shown as the mean SD and were analyzed by SPSS 26.0 software. One-way analysis of variance (ANOVA) was used to determine the statistical significance of the differences among groups, and a *P* value<0.05 was considered statistically significant.

## Results

### Cell proliferation assay of BMSCs

**Fig. 2:**
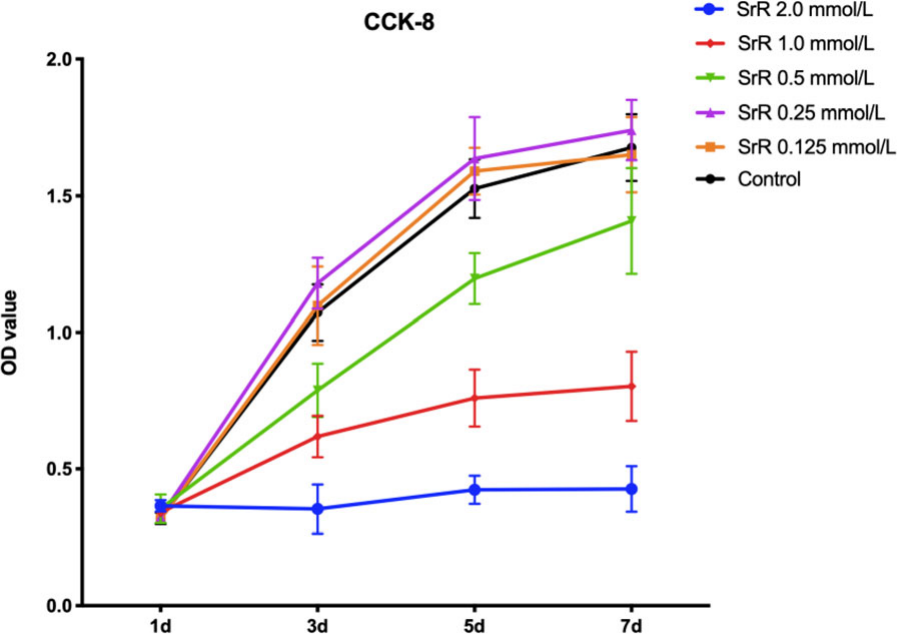
CCK-8 assays of the BMSCs treated with different concentrations of SrR

CCK-8 assays showed a clear dose-dependent effect of SrR on cell proliferation, and high concentrations (0.5, 1.0, and 2.0mmol/L) of SrR significantly suppressed cell viability at days 3, 5, and 7 (*P* <0.05). However, low concentrations (0.125, 0.25, and 0.5mmol/L) of SrR did not influence the proliferation of BMSCs at days 1, 3, 5, and 7, and no significant differences were observed between the control group and the 0.125 and 0.25mmol/L groups (Fig. 2).

### Alcian blue staining and toluidine blue staining

**Fig. 3:**
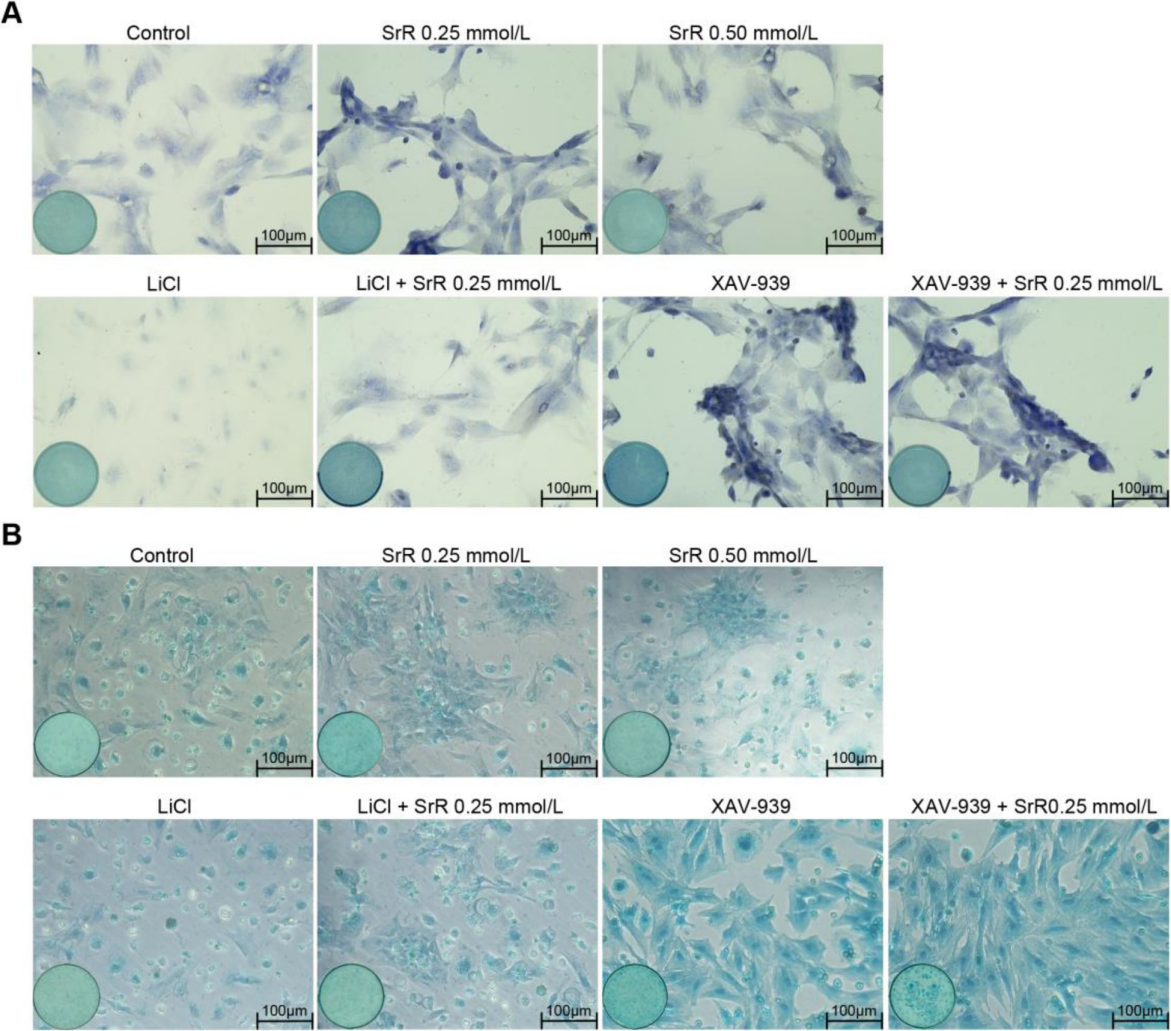
Toluidine blue staining (**a**) and alcian blue staining (**b**) of BMSCs under chondrogenic differentiation with 0.25mmol/L SrR and with or without LiCl or XAV-939. The result showed darker staining in the 0.25mmol/L SrR group than the control and 0.50mmol/L groups. The LiCl groups showed significantly weaker staining, while the LiCl plus SrR group showed darker staining. The XAV-939 and XAV-939 + SrR groups showed the darkest staining

Alcian blue staining showed the distribution and content of glycosaminoglycans (GAGs) produced by cells [25], and toluidine blue staining indicated the content of proteoglycans (PGs) [26]. Darker staining indicates better cell chondrogenesis. As shown in Fig. 3, a low concentration of SrR (0.25mmol/L) resulted in darker staining than that in the control group and the high concentration group (0.50mmol/L). LiCl treatment significantly suppressed chondrogenic differentiation; in this group, cells shrank or did not show any phenotypic changes and showed weak staining. LiCl plus SrR could improve the chondrogenic differentiation, resulting in darker staining than that in the LiCl group. XAV-939 is an inhibitor of -catenin but shows a strong chondrogenic effect. We found the darkest staining in the XAV-939 and XAV-939+SrR groups, with no major differences within the groups.

### Hyp assay

**Fig. 4:**
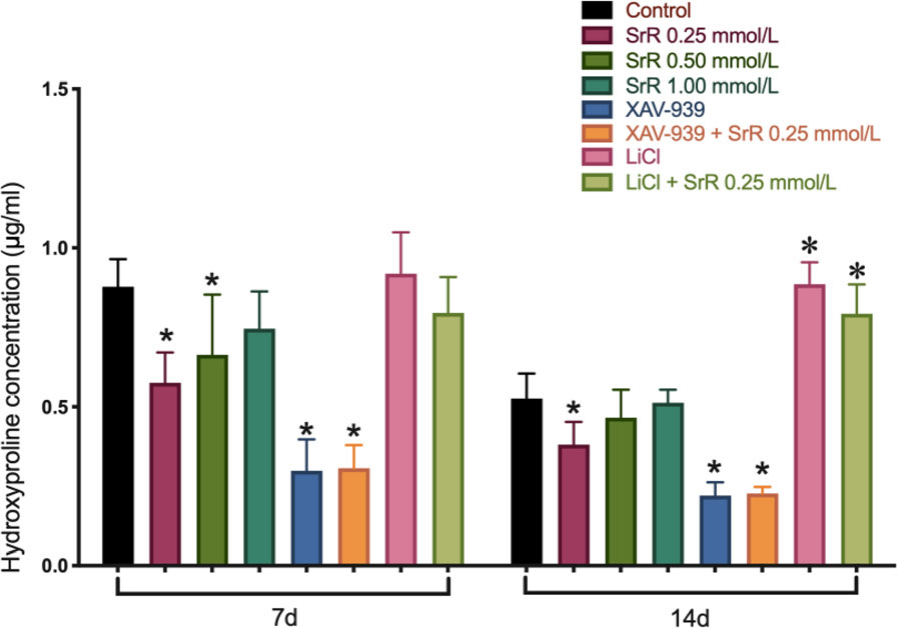
The result of Hyp assays. A significant decrease in Hyp content was detected in the 0.25mmol/L SrR group on days 7 and 14. The XAV-939 groups had the lowest secretion of Hyp, and SrR could slightly increase the Hyp content. In contrast, the LiCl groups showed the highest secretion of Hyp, and SrR could slightly suppress the Hyp content. *P <0.05 versus the control

Hyp is a characteristic amino acid and a degradation product of collagen. A high content of Hyp in the matrix indicates collagen degradation. We found a significantly lower content of Hyp in the 0.25mmol/L SrR group than in the control group and an increasing trend of Hyp with higher concentrations of SrR. XAV939 substantially suppressed the secretion of Hyp, while XAV-939 + SrR resulted in a slight but not significant increase in Hyp content. The LiCl groups had the opposite results: these groups had the highest content of Hyp, and SrR treatment suppressed this effect, without significant differences (Fig. 4).

### Wnt/-catenin signaling pathway analysis

**Fig. 5:**
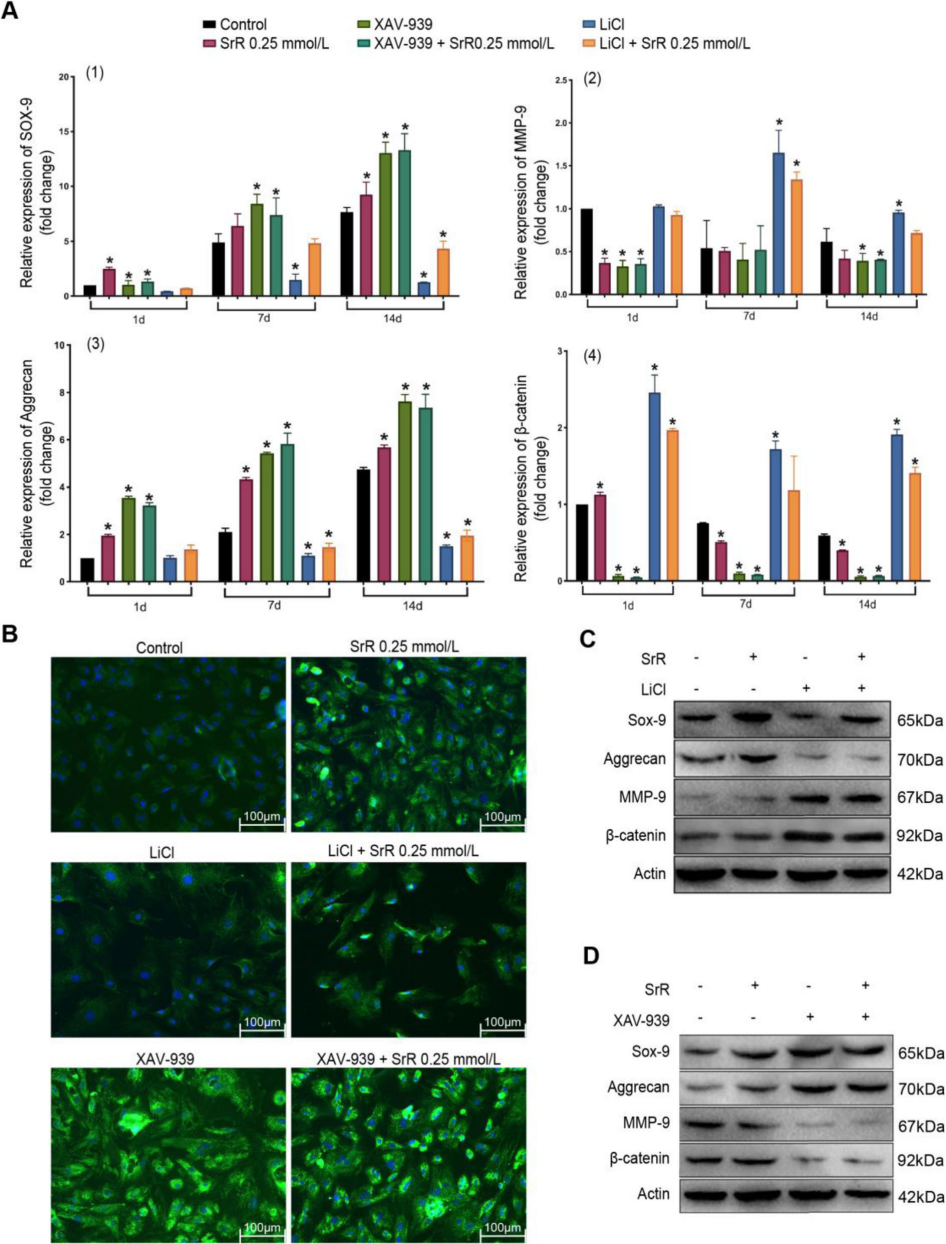
The results of Wnt/-catenin signaling pathway analysis. **a** The PCR assays of Sox-9 (1), MMP-9 (2), aggrecan (3), and -catenin (4). Data are presented as the meanSD. *P<0.05 versus the control. **b** The results of immunofluorescence staining of the Col-II protein. **c**, **d** show the results of WB assays. Sox-9 and aggrecan showed significantly higher expression and MMP-9 and -catenin showed significantly lower expression in the SrR treatment group than in the control group. XAV-939 significantly stimulated the synthesis of Sox-9 and aggrecan, inhibited MMP-9 and -catenin expression, and induced a similar trend when SrR was added. LiCl had the opposite effect: it downregulated Sox-9 and aggrecan expression and upregulated MMP-9 and -catenin expression, and SrR could also reverse these effects. The results were similar at both the gene and protein levels

We first tested the effect of -catenin inhibitors/agonists at the gene level. PCR assays revealed that inhibition of -catenin expression (XAV-939) increased the expression of Sox-9 and aggrecan, chondrogenic biomarkers, by 13.05-fold and 7.62-fold, respectively, on day 14 compared with that on the first day. Induction of -catenin (LiCl) decreased the expression of Sox-9 and aggrecan to 0.44-fold and 1.01-fold on day 1 and 1.27-fold and 1.50-fold on day 14, respectively; these values were much lower than those of the control group at each time point (*P* <0.05). The XAV-939 and XAV-939 + SrR groups did not show any significant differences within the groups. LiCl + SrR treatment significantly increased the expression of Sox-9 and aggrecan compared with that in the LiCl group. MMP-9 is negatively associated with these genes and causes matrix degradation. We observed the opposite trend for MMP-9 expression compared to Sox-9 and aggrecan expression. SrR significantly suppressed -catenin expression with LiCl treatment, but the expression of this molecule was not upregulated in the XAV-939 groups (Fig. 5a). Then, we performed protein assays. Immunofluorescence staining of the Col-II protein showed that the cells did not display any phenotypic changes after 14days of induction with the -catenin agonist LiCl, and LiCl plus SrR resulted in a stronger fluorescence intensity, which may indicate improved chondrogenic differentiation (Fig. 5b). WB assays also revealed that the protein expression showed a similar trend as the gene expression (Fig. 5c, d): SrR suppressed -catenin expression, thus increasing the protein expression of Sox-9 but not aggrecan.

### SrR-loaded silica nanospheres and GelMA gel

**Fig. 6:**
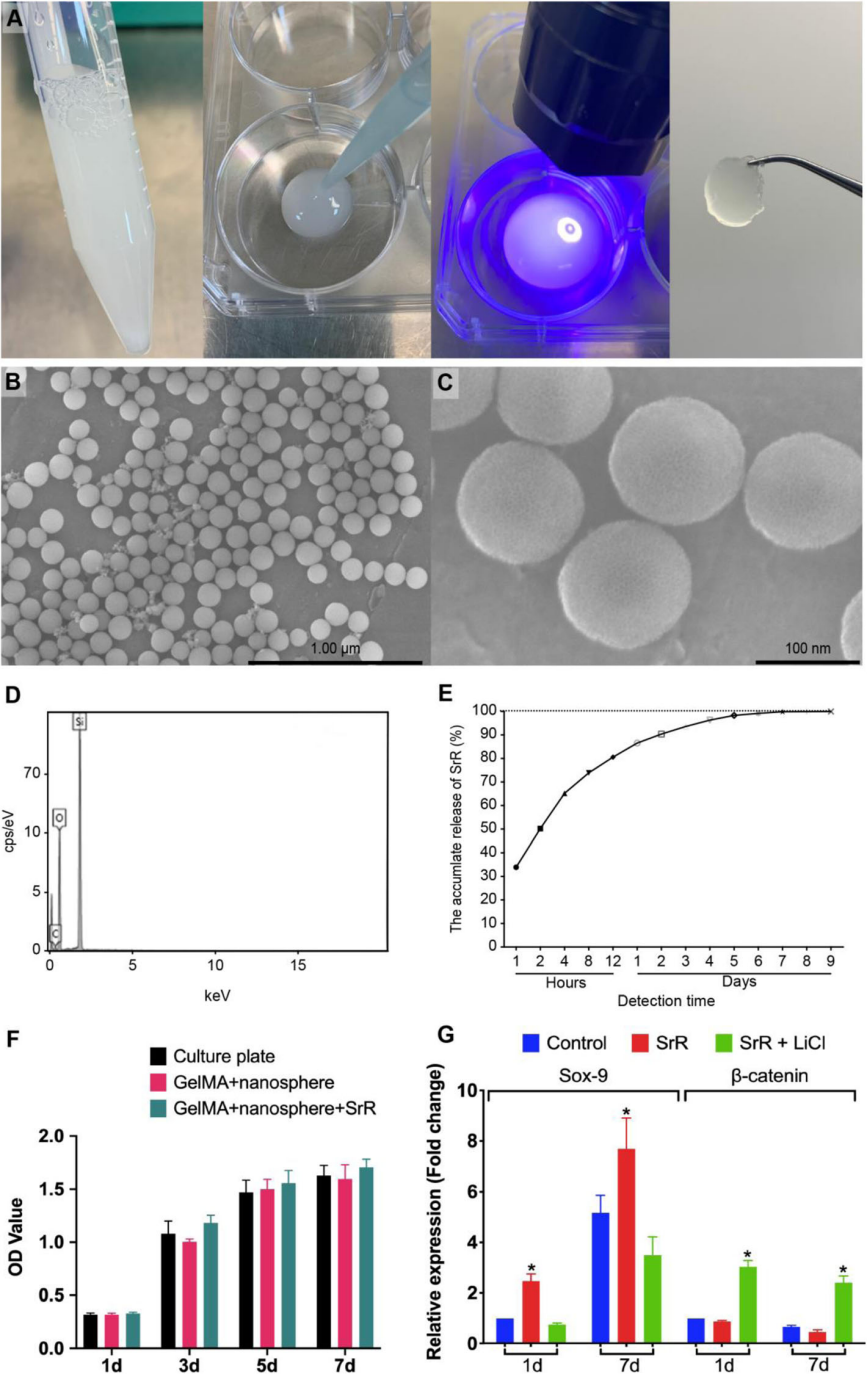
The GelMA gel and silica nanospheres. Gelatinous GelMA could be solidified by UV light (**a**). The silica nanospheres under SEM observation (**b**, **c**) and EDS analysis showed their pure silica content (**d**) and the controlled release of SrR for up to 7days (**e**). The silica nanospheres and GelMA did not influence the BMSCs cell viability (**f**), and the drug-loaded group had a significantly higher gene expression of Sox-9 and reduction of -catenin, while LiCl could significantly impress the expression of Sox-9 (**g**)

The average size of the silica nanospheres was 10020nm, with an inconspicuous hollow structure on the surface (Fig. 6b, c). The EDS analysis showed 53.26% O and 46.74% Si, similar to that of pure silica ingredients (Fig. 6d). The drug release test revealed an initial burst release of SrR on the first day and a sustained controlled release until complete release on day 7 (Fig. 6e). We mixed SrR-loaded silica nanospheres with UV-curved GelMA gel (Fig. 6a), and the degradation of the GelMA was very slow. It required 40days for complete absorption under the skin in nude mice [27], and we failed to observe any dissolution of GelMA in PBS liquid with changing the PBS every day for 14days. Therefore, we believed that the burst release of SrR from silica nanospheres did not matter, that the long-term controlled releasing of SrR in cartilage tissue could be achieved by the GelMA gel. The BMSCs showed a similar cell viability seeded on culture plates, GelMA + nanosphere and GelMA + SrR loaded nanospheres, which indicated a good biocompatibility (Fig. 6f). And the PCR assay confirmed a higher expression of Sox-9, and reduction of -catenin on drug-loaded scaffold (Fig. 6g). While culture medium with LiCl could significantly depress the expression of Sox-9, this also revealed that SrR promote the expression of Sox-9 by inhibition of -catenin.

### Histological observation

**Fig. 7:**
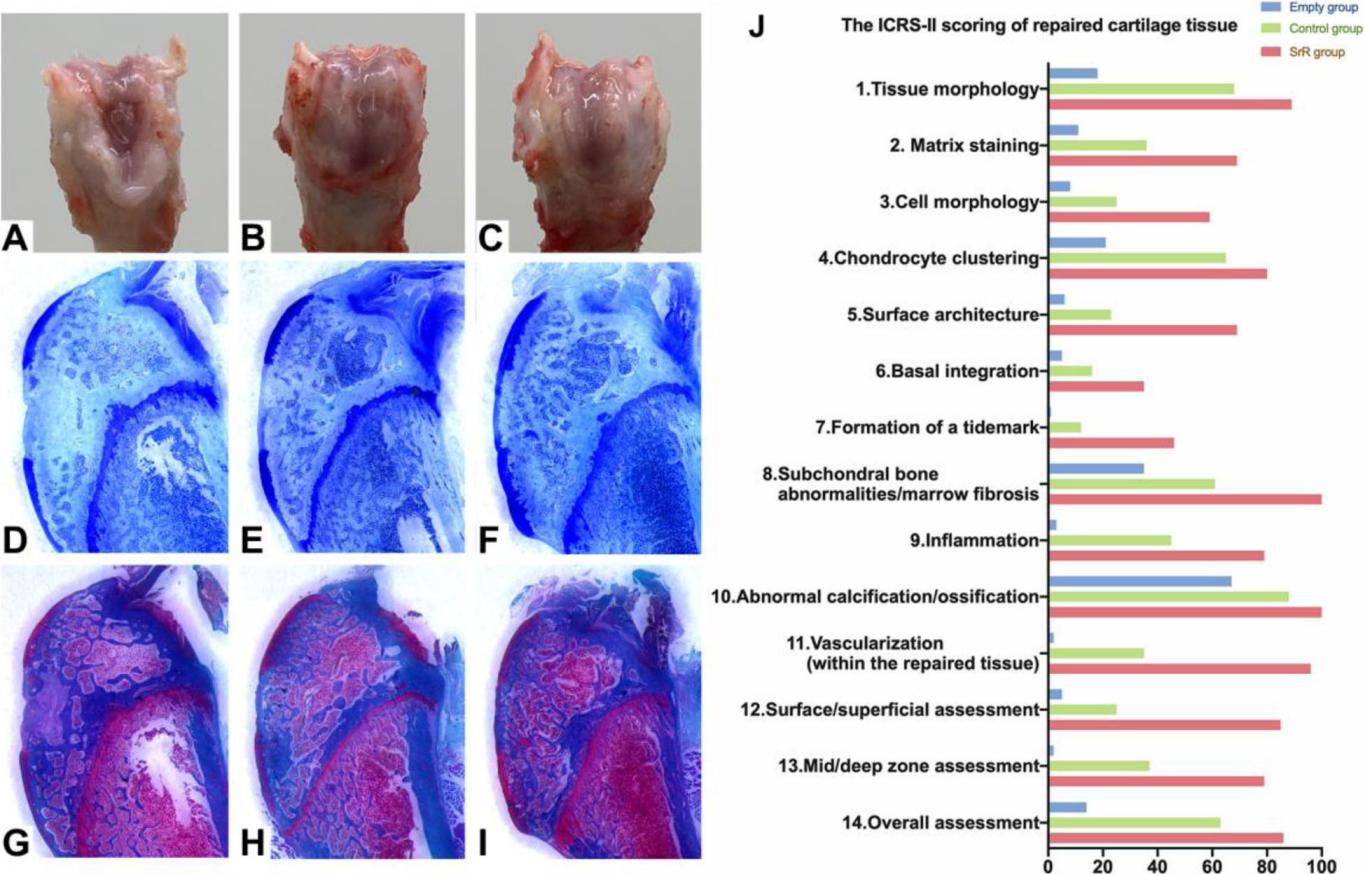
Histological observation. **a**, **d**, and **g** The empty group in which the wound was not filled; **b**, **e**, and **h** the control group in which the wound was filled with silica nanospheres plus GelMA gel; and **c**, **f**, and **i** show the SrR group in which the wound was filled with SrR-loaded silica nanospheres plus GelMA gel. Toluidine blue staining (**d**, **e**, and **g**) and safranin O/fast green staining (**g**, **h**, and **i**) revealed that the SrR group had a better cartilage tissue repair effect and significantly higher ICRS-II scores than the other two groups (**j**). There were significant differences of ICRS-II scores between the empty group, control group, and SrR group (*P*<0.05)

After 12weeks of healing, we sacrificed all of the rats and observed obvious tissue repair in the SrR treatment group compared with the empty group and the control group by the naked eye (Fig. 7ac). Figure 7a, d, and g shows the empty group in which the wound was not filled; Fig. 7b, e, and Hs show the control group with silica nanospheres plus GelMA gel; and Fig. 7c, f, and i shows the SrR group with SrR-loaded silica nanospheres plus GelMA gel. Toluidine blue staining (Fig. 7d, e, and g) and safranin O/fast green staining (Fig. 7gi) revealed that the SrR group had a better cartilage repair effect and significantly higher ICRS-II scores than the other two groups (Fig. 7j). The cartilage defect with a diameter of 2.5mm could not be self-healed, as shown by the empty group in which the fibrous tissues had many vessels inside but no hyaline cartilage. GelMA gel with silica nanospheres could significantly induce cartilage regeneration. Basal integration and a smooth surface with different levels of chondrification could be found in the control group and the SrR group, but the SrR group had a better performance than the control group. And there were more subchondral bone abnormalities or abnormal calcification around the defect area in the empty group.

### Immunofluorescent and immunohistochemical analysis

**Fig. 8:**
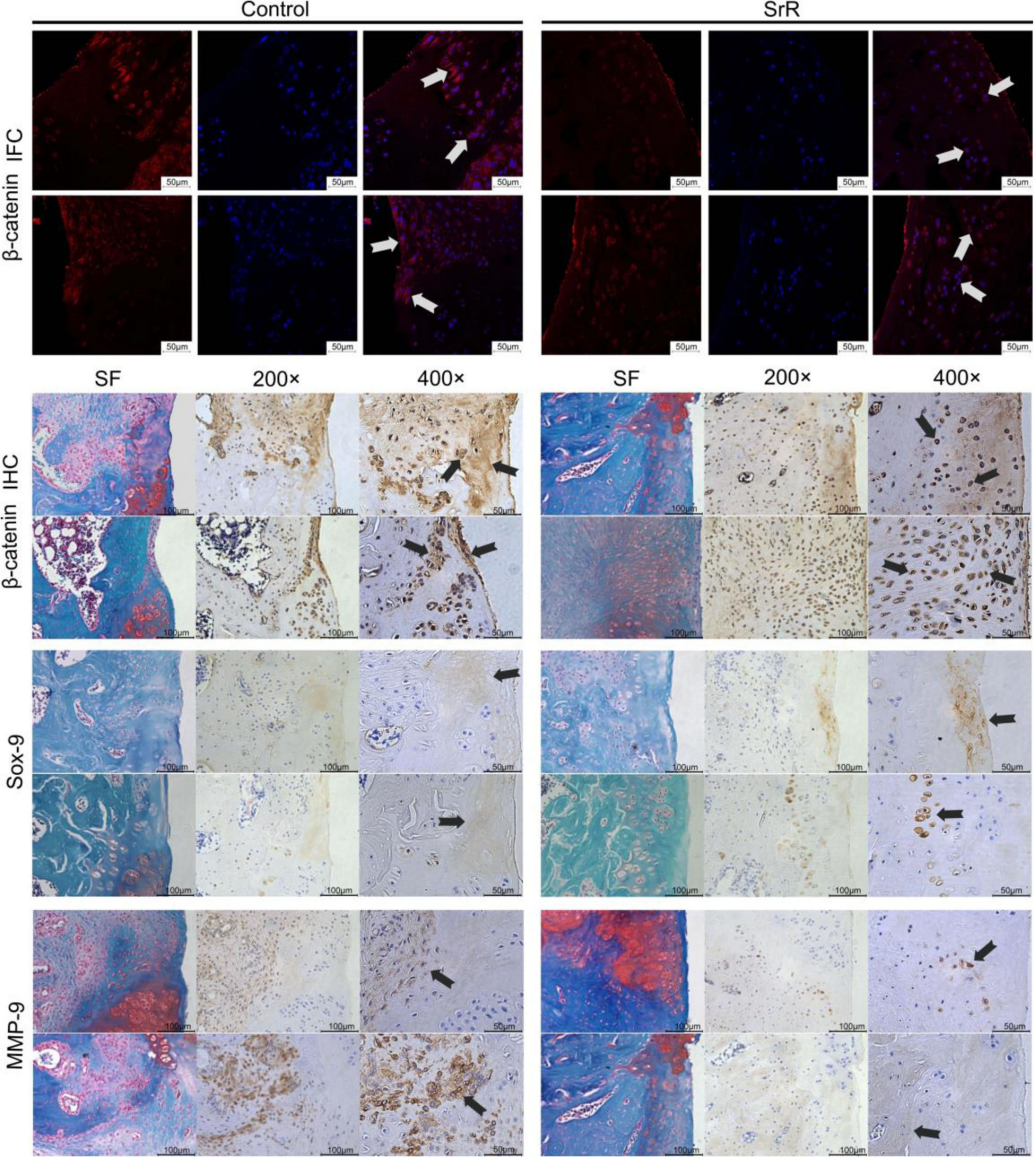
IFC/IHC staining of -catenin, SF staining, and IHC staining of Sox-9 and MMP-9 were performed. The arrows show the accumulation and nuclear translocation of -catenin in the control group, and less stained in the SrR group, the staining of -catenin in fibrous tissue, chondrocytes, and the frontier of chondrification, the staining of Sox-9 in newly formed cartilage tissue and chondrocytes, and the staining of MMP-9 in the inflammatory area

Immunofluorescent (IFC) staining showed the localization and content of -catenin. -catenin accumulate and translocate into the nucleus is the determined step of activation, and we found an obvious stronger stain and nuclear translocation of -catenin in the control group than the SrR group. Immunohistochemistry (IHC) staining and safranin O/fast green (SF) staining showed a clearer view of the relationship of -catenin, Sox-9, MMP-9, and the new regenerated cartilage tissue. -catenin accumulated in fibrous tissue and in blood vessels, chondrocytes were darkly stained, and the basal line of the cartilage layer may be the frontier of chondrification, while mature cartilage was not stained. Sox-9 was darker stained in SrR group, and mainly gathered in newly formed cartilage and chondrocytes. MMP-9 was stained in the inflammatory area, and the normal cartilage area was not stained (Fig. 8).

## Discussion

SrR not only inhibited subchondral bone resorption but also directly promoted chondrogenic differentiation of stem cells. Our results showed that SrR significantly promoted the contents of GAGs and PGs and increased the expression of cartilage marker genes and proteins such as Sox-9, Col-II, and aggrecan in chondrogenic differentiation medium. These results verified the conclusions of previous studies. As early as 1985, Reinholt found that Sr ions could stimulate cartilage regeneration and enhance the aggregation of PGs and hyaluronic acid, thus inducing chondrocytes to produce extracellular matrix in the epiphyseal area [28]. Henrotin treated chondrocytes from OA patients with SrR and found that SrR could strongly stimulate PG synthesis, promote the activity of insulin-like growth factor-1 (IGI-I), and effectively inhibit the expression of MMPs [29]. Yu found that SrR could also increase the synthesis of type II collagen and PGs by upregulating the expression of Sox-9, a biomarker of chondrogenic differentiation [30]. These results all confirmed the role of SrR in promoting chondrogenesis at the cellular level.

Chondrogenic differentiation of stem cells is a complex procedure. The Wnt signaling pathway plays an important role in cell proliferation, migration, and also the chondrogenic differentiation. The mechanism of the Wnt pathway in chondrogenic differentiation is complicated, involves many bidirectionally regulated factors, and varies at different times [31, 32]. Among these pathways, the canonical Wnt pathway has been relatively well studied. -catenin is the key controlling element of the canonical Wnt pathway. The signaling pathway triggers frizzled protein (FZD) and low-density lipoprotein receptor-related protein 5/6 (LRP5/6) to phosphorylate disheveled protein (DVL) to inhibit glycogen synthase kinase-3 (GSK-3). The inhibition of GSK-3 induces -catenin disassociation from adenomatosis polyposis coli (APC), Axin, and GSK-3. Then, unphosphorylated -catenin accumulates in the cytoplasm and transfers to the nucleus, where it acts as a coactivator of the Tcf/Lef transcription factor to regulate the transcription of downstream genes [33,35]. The canonical Wnt/-catenin pathway has a negative regulatory effect on chondrogenesis. Many studies have confirmed that -catenin can inhibit Sox-9 expression while promoting Runx-2 expression. When the Wnt/-catenin pathway is activated, stem cells show a trend of osteogenic differentiation, while inhibition of the Wnt/-catenin pathway will lead to chondrogenic differentiation, even if cells are cultured in osteogenic induction medium [36,39]. Negative functional interactions between -catenin and Sox-9 were reported by Akiyama H, and the inhibitory effect between -catenin and Sox-9 was reciprocal. On the one hand, -catenin binds to the Sox-9 transactivation domain and induces Sox-9 degradation; on the other hand, Sox-9 represses -catenin/Tcf/Lef complex activities. There are -catenin binding sites on the surface of Sox-9 protein, and its sequence is similar of that on Tcf-lef, thus the formation of the Sox-9/-catenin complex could competitively inhibit the binding of Tcf/Lef and -catenin. Sox-9/-catenin complex leads to the degradation of -catenin through the ubiquitination/26S proteasome pathway [40]. In the present study, XAV-939, which targets the tankyrase enzyme, was used as an antagonist of -catenin [41], and the results showed a significant promotion of chondrogenic differentiation. LiCl promotes the accumulation of -catenin by inhibiting GSK-3, which was used as an agonist of -catenin [42], and inhibition of chondrogenic differentiation was observed. These results confirmed the negative regulatory effect of the Wnt/-catenin pathway on chondrogenic differentiation. There were also suggestions indicated that the Wnt/-catenin pathway was suppressed only in the early stage of stem cell chondrogenic differentiation (within 21days) and was not inhibited beyond this period [43]. This phenomenon was not observed in our study because we only did 14days of induction.

Our study revealed the role of SrR in promoting chondrogenic differentiation by inhibiting -catenin. We found that adding a low concentration (0.25mmol/L) of SrR to the chondrogenic induction solution could inhibit the expression of -catenin and increase the expression of Sox-9, Col-II, aggrecan, and other chondrogenic biomarker proteins. -catenin agonists significantly inhibited chondrogenic differentiation of BMSCs, while SrR relieved this inhibition. Inhibition of -catenin significantly promoted chondrogenic differentiation, while SrR addition did not further enhance this effect but also did not suppress it. These results suggested that SrR promoted the chondrogenic differentiation of stem cells by inhibiting -catenin. Theoretically, Sr has a similar molecular structure and physiological function as calcium. As described before, SrR could activate the Ca^2+^ receptor on the cell membrane surface, enhanced the expression of Wnt3a through the Cn-NFAT pathway, and then activated the canonical Wnt/-catenin pathway. SrR can also increase the expression of Wnt5a, the FZD receptor family, and Ror2/Ryk coreceptors and regulate the downstream RhoA-, JNK- and Ca^2+^-dependent signaling pathways through the noncanonical Wnt pathway to promote the proliferation and differentiation of osteoblast cells [16]. Our results showed that SrR could inhibit -catenin, thus the canonical Wnt pathway in a chondrogenic induction environment. But, whether SrR acts directly on -catenin or through the Cn-NFAT pathway still needs further investigation.

The results of the in vivo study confirmed that the local release of SrR has a superior effect in promoting cartilage regeneration. As shown by toluidine blue and safranin O/fast green staining, in the empty group without any scaffold, their cartilage defect areas were filled with fibrous tissue, and no cartilage regeneration could be observed. This finding indicates that the round cartilage defect with a diameter of 2.5mm could not be self-healed in rats. While, notable cartilage regeneration was observed with GelMA filling, and a much better regenerative effect was observed in the SrR-loaded GelMA group, which had higher ICRS-II scores than other groups. IFC staining revealed a higher activation level of -catenin in the control group than SrR treated group, that -catenin was accumulated in the cytoplasm and translocated into the nucleus. And IHC staining showed a clearer view of -catenin positions that it was intensely stained in prehypertrophic chondrocytes and periosteal cells and in blood cells but weaker or no stained in mature cartilage tissue, and control group were darker stained than the SrR group. These results indicated a weaker -catenin activation level of SrR treatment in vivo, and a better cartilage regeneration effect. Sox-9 is the biomarker of cartilage forming and was stained more intensively in the front line of chondrification in the SrR group. MMP-9 is an inflammatory factor that accumulates in fibrous tissue. Weaker -catenin and MMP-9 staining were observed in the area with better cartilage regeneration. The application of drugs has been a prospective treatment option for cartilage tissue engineering in OA. Drugs, as pure chemicals, show no immunogenicity, are relatively safe, are easily obtained, and can be easily stored and used [6]. Our results showed that SrR could be used as a drug for cartilage tissue engineering and has a good effect on cartilage regeneration.

## Conclusions

SrR could promote BMSC chondrogenic differentiation by inhibiting the Wnt/-catenin signaling pathway and accelerate cartilage regeneration in rat femoral condyle defects. SrR could be an alternative drug option for cartilage tissue engineering.

## Data Availability

All data generated or analyzed during this study are included in this published article.

## Abbreviations

APC: Adenomatosis polyposis coli
BMSCs: Bone mesenchymal stem cells
Cn-NFAT: Calcineurin-activated T nuclear factor
CTAB: Cetyltrimethylammonium bromide
DMOAD: Disease-modifying osteoarthritis drug
DVL: Disheveled protein
EDS: Energy dispersive spectrum
FZD: Frizzled protein
GAGs: Glycosaminoglycans
GelMA: Gelatine methacryloyl
GSK-3: Glycogen synthase kinase-3
Hyp: Hydroxyproline
ICRS-II: International Cartilage Repair Society - II scoring system
IFC: Immunofluorescent staining
IGI-I: Insulin-like growth factor-1
IHC: Immunohistochemical staining
LAP: Lithium phenyl-2,4,6-trimethylbenzoylphosphinate
LRP5/6: Low-density lipoprotein receptor-related protein 5/6
MMPs: Matrix metalloproteinases
MSNs: Mesoporous silica nanoparticles
PGs: Proteoglycans
SD rat: SpragueDawley rat
SEKIOA: Strontium Ranelate Efficacy in Knee Osteoarthritis
SEM: Scanning electron microscopy
SrR: Strontium ranelate
TEOS: Tetraethoxysilane
TMJ-OA: Temporomandibular joint osteoarthritis

## References

[1] Bernhardt O, Biffar R, Kocher T, Meyer G (2007). Prevalence and clinical signs of degenerative temporomandibular joint changes validated by magnetic resonance imaging in a non-patient group. Ann Anat.

[2] Shoukri B, Prieto JC, Ruellas A, Yatabe M, Sugai J, Styner M (2019). Minimally invasive approach for diagnosing TMJ osteoarthritis. J Dent Res.

[3] Wang XD, Zhang JN, Gan YH, Zhou YH (2015). Current understanding of pathogenesis and treatment of TMJ osteoarthritis. J Dent Res.

[4] Bijlsma JW, Berenbaum F, Lafeber FP (2011). Osteoarthritis: an update with relevance for clinical practice. Lancet.

[5] Alexandersen P, Karsdal MA, Qvist P, Reginster JY, Christiansen C (2007). Strontium ranelate reduces the urinary level of cartilage degradation biomarker CTX-II in postmenopausal women. Bone.

[6] Roseti L, Desando G, Cavallo C, Petretta M, Grigolo B (2019). Articular Cartilage Regeneration in Osteoarthritis. Cells.

[7] Zhang S, Yap AU, Toh WS (2015). Stem Cells for Temporomandibular Joint Repair and Regeneration. Stem Cell Rev Rep.

[8] Reginster JY, Badurski J, Bellamy N, Bensen W, Chapurlat R, Chevalier X (2013). Efficacy and safety of strontium ranelate in the treatment of knee osteoarthritis: results of a double-blind, randomised placebo-controlled trial. Ann Rheum Dis.

[9] Han W, Fan S, Bai X, Ding C (2017). Strontium ranelate, a promising disease modifying osteoarthritis drug. Expert Opin Investig Drugs.

[10] Pelletier JP, Roubille C, Raynauld JP, Abram F, Dorais M, Delorme P (2015). Disease-modifying effect of strontium ranelate in a subset of patients from the Phase III knee osteoarthritis study SEKOIA using quantitative MRI: reduction in bone marrow lesions protects against cartilage loss. Ann Rheum Dis.

[11] Tenti S, Cheleschi S, Guidelli GM, Galeazzi M, Fioravanti A (2014). What about strontium ranelate in osteoarthritis? Doubts and securities. Mod Rheumatol.

[12] Rodrigues TA, Freire AO, Bonfim BF, Cartgenes MSS, Garcia JBS (2018). Strontium ranelate as a possible disease-modifying osteoarthritis drug: a systematic review. Braz J Med Biol Res.

[13] Marie PJ, Felsenberg D, Brandi ML (2011). How strontium ranelate, via opposite effects on bone resorption and formation, prevents osteoporosis. Osteoporos Int.

[14] Tat SK, Pelletier JP, Mineau F, Caron J, Martel-Pelletier J (2011). Strontium ranelate inhibits key factors affecting bone remodeling in human osteoarthritic subchondral bone osteoblasts. Bone.

[15] Pelletier JP, Kapoor M, Fahmi H, Lajeunesse D, Blesius A, Maillet J (2013). Strontium ranelate reduces the progression of experimental dog osteoarthritis by inhibiting the expression of key proteases in cartilage and of IL-1 in the synovium. Ann Rheum Dis.

[16] Fromigu O, Ha E, Barbara A, Marie PJ (2010). Essential role of nuclear factor of activated T cells (NFAT)-mediated Wnt signaling in osteoblast differentiation induced by strontium ranelate. J Biol Chem.

[17] Qiao Y, Liu X, Zhou X, Zhang H, Zhang W, Xiao W (2020). Gelatin Templated Polypeptide Co-Cross-Linked Hydrogel for Bone Regeneration. Adv Healthc Mater.

[18] Chen L, Zhou X, He C (2019). Mesoporous silica nanoparticles for tissue-engineering applications. Wiley Interdiscip Rev Nanomed Nanobiotechnol.

[19] Guo X, Wei S, Lu M, Shao Z, Lu J, Xia L (2016). Dose-dependent Effects of Strontium Ranelate on Ovariectomy Rat Bone Marrow Mesenchymal Stem Cells and Human Umbilical Vein Endothelial Cells. Int J Biol Sci.

[20] Solchaga LA, Penick KJ, Welter JF (2011). Chondrogenic differentiation of bone marrow-derived mesenchymal stem cells: tips and tricks. Methods Mol Biol.

[21] Zhang Y, Chang M, Bao F, Xing M, Wang E, Xu Q (2019). Multifunctional Zn doped hollow mesoporous silica/polycaprolactone electrospun membranes with enhanced hair follicle regeneration and antibacterial activity for wound healing. Nanoscale.

[22] Yue K, Trujillo-de Santiago G, Alvarez MM, Tamayol A, Annabi N, Khademhosseini A (2015). Synthesis, properties, and biomedical applications of gelatin methacryloyl (GelMA) hydrogels. Biomaterials.

[23] Takahashi I, Hoso M, Matsuzaki T (2012). Histopathological Effects of Loading on Cartilage Repair in a Rat Full-thickness Articular Cartilage Defect Model. J Phys Ther Sci.

[24] Mainil-Varlet P, Van Damme B, Nesic D, Knutsen G, Kandel R, Roberts S (2010). A new histology scoring system for the assessment of the quality of human cartilage repair: ICRS II. Am J Sports Med.

[25] Irani S, Honarpardaz A, Choubini N, Pezeshki-Modaress M, Zandi M (2020). Chondro-inductive nanofibrous scaffold based gelatin/polyvinylalcohol/chondroitin sulfate for cartilage tissue engineering. Polym Adv Technol.

[26] Bergholt NL, Lysdahl H, Lind M, Foldager CB (2019). A Standardized Method of Applying Toluidine Blue Metachromatic Staining for Assessment of Chondrogenesis. Cartilage.

[27] Wang Z, An G, Zhu Y, Liu X, Chen Y, Wu H (2019). 3D-printable self-healing and mechanically reinforced hydrogels with host-guest non-covalent interactions integrated into covalently linked networks. Mater Horiz.

[28] Reinholt FP, Engfeldt B, Heinegrd D, Hjerpe A (1985). Proteoglycans and glycosaminoglycans of normal and strontium rachitic epiphyseal cartilage. Coll Relat Res.

[29] Henrotin Y, Labasse A, Zheng SX, Galais P, Tsouderos Y, Crielaard JM (2001). Strontium ranelate increases cartilage matrix formation. J Bone Miner Res.

[30] Yu DG, Ding HF, Mao YQ, Liu M, Yu B, Zhao X (2013). Strontium ranelate reduces cartilage degeneration and subchondral bone remodeling in rat osteoarthritis model. Acta Pharmacol Sin.

[31] Monteagudo S, Lories RJ (2017). Cushioning the cartilage: a canonical Wnt restricting matter. Nat Rev Rheumatol.

[32] Lories RJ, Corr M, Lane NE (2013). To Wnt or not to Wnt: the bone and joint health dilemma. Nat Rev Rheumatol.

[33] Clevers H (2006). Wnt/beta-catenin signaling in development and disease. Cell..

[34] Clevers H, Nusse R (2012). Wnt/-catenin signaling and disease. Cell.

[35] Nusse R, Clevers H (2017). Wnt/-Catenin Signaling, Disease, and Emerging Therapeutic Modalities. Cell.

[36] Fang M, Alfieri CM, Hulin A, Conway SJ, Yutzey KE (2014). Loss of -catenin promotes chondrogenic differentiation of aortic valve interstitial cells. Arterioscler Thromb Vasc Biol.

[37] Day TF, Guo X, Garrett-Beal L, Yang Y (2005). Wnt/beta-catenin signaling in mesenchymal progenitors controls osteoblast and chondrocyte differentiation during vertebrate skeletogenesis. Dev Cell.

[38] Hill TP, Spter D, Taketo MM, Birchmeier W, Hartmann C (2005). Canonical Wnt/beta-catenin signaling prevents osteoblasts from differentiating into chondrocytes. Dev Cell.

[39] Luo S, Shi Q, Zha Z, Yao P, Lin H, Liu N (2013). Inactivation of Wnt/-catenin signaling in human adipose-derived stem cells is necessary for chondrogenic differentiation and maintenance. Biomed Pharmacother.

[40] Akiyama H, Lyons JP, Mori-Akiyama Y, Yang X, Zhang R, Zhang Z (2004). Interactions between Sox9 and beta-catenin control chondrocyte differentiation. Genes Dev.

[41] Huang SM, Mishina YM, Liu S, Cheung A, Stegmeier F, Michaud GA (2009). Tankyrase inhibition stabilizes axin and antagonizes Wnt signalling. Nature..

[42] Clment-Lacroix P, Ai M, Morvan F, Roman-Roman S, Vayssire B, Belleville C (2005). Lrp5-independent activation of Wnt signaling by lithium chloride increases bone formation and bone mass in mice. Proc Natl Acad Sci U S A.

[43] Im GI, Quan Z (2010). The effects of Wnt inhibitors on the chondrogenesis of human mesenchymal stem cells. Tissue Eng Part A.

